# Supplementary material for: LuxT controls specific quorum-sensing-regulated behaviors in Vibrionaceae spp. via repression of qrr1, encoding a small regulatory RNA
Source: PLoS Genet. 2021 Apr 1;17(4):e1009336. doi: 10.1371/journal.pgen.1009336 (PMC8043402; doi:10.1371/journal.pgen.1009336)
Supplement: S1 Table — (PDF) [file pgen.1009336.s001.pdf]

**S1 Table. Strains used in this study**

(WT strains are depicted in bold with variants listed below them.)

| Strain | Relevant genotype or feature | Reference |
|---|---|---|
| ***V. harveyi* BB120** | WT | [1] |
| ME287 | Δ*luxT* | This study |
| ME790 | *aphA-3xFLAG* | This study |
| ME791 | *aphA-3xFLAG* Δ*luxT* | This study |
| ME792 | *3xFLAG-luxR* | This study |
| ME793 | *3xFLAG-luxR* Δ*luxT* | This study |
| JSV780 | *luxO* D61E | [2] |
| ME181 | *luxO* D61E Δ*luxT* | This study |
| KT39 | Δ*qrr*1 | [3] |
| ME431 | Δ*qrr*1 Δ*luxT* | This study |
| KT282 | Δ*qrr*1-5 | [3] |
| ME794 | Δ*qrr*1-5 Δ*luxT* | This study |
| BB721 | Δ*luxO* | [4] |
| ME393 | Δ*luxO* Δ*luxT* | This study |
| ME291 | Δ*VIBHAR_RS03920* | This study |
| ME302 | Δ*VIBHAR_RS03920* Δ*luxT* | This study |
| ME795 | *luxO* D61E Δ*qrr*1 | This study |
| ME796 | *luxO* D61E Δ*qrr*1 Δ*luxT* | This study |
| BH421 | *luxA*::Tn*5* | [5] |
| ME911 | *luxA*::Tn*5* Δ*luxT* | This study |
| ***Vibrio cholerae* C6706** | WT | [6] |
| ME651 | Δ*luxT* | This study |
| SLS340 | *luxO* D61E | [7] |
| ME797 | *luxO* D61E Δ*luxT* | This study |
| ***V. parahaemolyticus* BB22OP** | WT | [8] |
| ME798 | Δ*swrT* | This study |
| ME799 | *luxO* D61E | This study |
| ME800 | *luxO* D61E Δ*swrT* | This study |
| ***A. fischeri* ES114** | WT | [9] |
| ME226 | Δ*luxT* | [10] |
| ME801 | *luxO* D55E | This study |
| ME802 | *luxO* D55E Δ*luxT* | This study |
| ME803 | *luxO* D55E Δ*qrr*1 | This study |
| ME804 | *luxO* D55E Δ*qrr*1 Δ*luxT* | This study |
| ***E. coli* S17-1 λ*pir*** | WT | [11] |
| ***E. coli* BL21 (DE3)** | *E. coli str. B, $F^-$ ompT hsdSB ($rBmB^-$) gal dcm (DE3)* | Agilent |
| ***E. coli* BW25113** | *$lacI^q$ $rrnB_{T14}$ Δ$lacZ_{WJ16}$ hsdR514 Δar $aBAD_{AH33}$ Δ$rhaBAD_{LD78}$* | [12] |

**Table S1 References**


1. Bassler BL, Greenberg EP, Stevens AM. Cross-species induction of luminescence in the quorum-sensing bacterium *Vibrio harveyi*. J Bacteriol. 1997 Jun;179(12):4043–5.

2. Freeman JA, Bassler BL. A genetic analysis of the function of LuxO, a two-component response regulator involved in quorum sensing in *Vibrio harveyi*. Mol Microbiol. 1999 Jan;31(2):665–77.

3. Tu KC, Bassler BL. Multiple small RNAs act additively to integrate sensory information and control quorum sensing in *Vibrio harveyi*. Genes Dev. 2007 Jan 15;21(2):221–33.

4. Henke JM, Bassler BL. Quorum sensing regulates type III secretion in *Vibrio harveyi* and *Vibrio parahaemolyticus*. J Bacteriol. 2004 Jun;186(12):3794–805.

5. Waters CM, Wu JT, Ramsey ME, Harris RC, Bassler BL. Control of the type 3 secretion system in *Vibrio harveyi* by quorum sensing through repression of ExsA. Appl Environ Microbiol. 2010 Aug;76(15):4996–5004.

6. Thelin KH, Taylor RK. Toxin-coregulated pilus, but not mannose-sensitive hemagglutinin, is required for colonization by *Vibrio cholerae* O1 El Tor biotype and O139 strains. Infect Immun. 1996 Jul;64(7):2853–6.

7. Waters CM, Lu W, Rabinowitz JD, Bassler BL. Quorum sensing controls biofilm formation in *Vibrio cholerae* through modulation of cyclic di-GMP levels and repression of vpsT. J Bacteriol. 2008 Apr;190(7):2527–36.

8. McCarter LL. OpaR, a homolog of *Vibrio harveyi* LuxR, controls opacity of *Vibrio parahaemolyticus*. J Bacteriol. 1998 Jun;180(12):3166–73.

9. Boettcher KJ, Ruby EG. Depressed light emission by symbiotic *Vibrio fischeri* of the sepiolid squid *Euprymna scolopes*. J Bacteriol. 1990 Jul;172(7):3701–6.

10. Eickhoff MJ, Bassler BL. *Vibrio fischeri* siderophore production drives competitive exclusion during dual-species growth. Mol Microbiol. 2020 Aug;114(2):244–61.

11. de Lorenzo V, Timmis KN. Analysis and construction of stable phenotypes in gram-negative bacteria with Tn5- and Tn10-derived minitransposons. Methods Enzymol. 1994;235:386–405.

12. Datsenko KA, Wanner BL. One-step inactivation of chromosomal genes in *Escherichia coli* K-12 using PCR products. Proc Natl Acad Sci USA. 2000 Jun 6;97(12):6640–5.
