# Supplementary material for: LuxT controls specific quorum-sensing-regulated behaviors in Vibrionaceae spp. via repression of qrr1, encoding a small regulatory RNA
Source: PLoS Genet. 2021 Apr 1;17(4):e1009336. doi: 10.1371/journal.pgen.1009336 (PMC8043402; doi:10.1371/journal.pgen.1009336)
Supplement: S2 Table — (PDF) [file pgen.1009336.s002.pdf]

S2 Table. Oligonucleotides used in this study
Sequences are provided in the 5' → 3' direction

Name	Sequence	Description
ME-633	GGTATATCTCCTTCTTAAAGTTAAACAAAATTAT T	Plasmid construction, pET15b cloning
ME-690	GCAGCTGCGCATCATCATCATCATCACTAACAA AGCCCGAAAGGAAGCTG	Plasmid construction, pET15b-*6xHis* cloning
ME-635	TTTTGTTTAACTTTAAGAAGGAGATATACCATG CCAAAGCGTAGTAAAGAAGATAC	Plasmid construction, pET15b-*luxT-6xHis*
ME-691	TTAGTGATGATGATGATGATGCGCAGCTGCTTT GCTCATTTGAATTAACGAACG	Plasmid construction, pET15b-*luxT-6xHis*
ME-924	GTGCCATAATTTAACCTGTTGATATTCG	P_{luxO} EMSA probe forward primer
ME-708	TCGCATTACGCTTTGCATTTTG	P_{luxO} EMSA probe reverse primer
ME-920	ATGGCGCTTTGCCTGGTTTC	$P_{Control}$ EMSA probe forward primer (from *E. coli* MG1655)
ME-927	TTGAGGGGACGACGACAGTATC	$P_{Control}$ EMSA probe reverse primer (from *E. coli* MG1655)
pRE112-F	ATGCAGTTCACTTACACCGCTTC	Plasmid construction, pRE112 mediated chromosomal alterations
pRE112-R	GGGATCGGGCCCTATCACTT	Plasmid construction, pRE112 mediated chromosomal alterations
ME-121	GGGTTGAGAAGCGGTGTAAGTGAACTGCATTG TGCTACTGATGTGTACCGATG	Plasmid construction, *luxT* deletion (*V. harveyi)*
ME-122	TGGCATATTTTAAGCTCTTCTCTTTG	Plasmid construction, *luxT* deletion (*V. harveyi)*
ME-123	TTTACAAAGAGAAGAGCTTAAAATATGCCATAA CACTAGAACAAGAAAGCCCCG	Plasmid construction, *luxT* deletion (*V. harveyi)*
ME-124	ACGCCTGAATAAGTGATAGGGCCCGATCCCGT ACACTGCTTCCTATCTCAGC	Plasmid construction, *luxT* deletion (*V. harveyi)*
STR-0040	CGTGAGCGTATCCCGGTATCTAT	qRT-PCR, *hfq* (*V. harveyi)*
STR-0041	TTGCAGTTTGATACCGTTCACAAG	qRT-PCR, *hfq* (*V. harveyi)*
ME-255	ACAGCCCATGGTTCTATCGATAC	qRT-PCR, *luxO* (*V. harveyi)*
ME-256	CTTTACGGATCGCATTGTTCACC	qRT-PCR, *luxO* (*V. harveyi)*
ME-416	TATACAACAGGGCAGCGTTGG	qRT-PCR, *luxC* (*V. harveyi)*

ME-417	TCCAATTTGCTTCGAGTTTCGC	qRT-PCR, *luxC* (*V. harveyi)*
ME-871	GGGTTGAGAAGCGGTGTAAGTGAACTGCATTTAGAAGAAGCATTGATGGTGACG	Plasmid construction, pRE112-*aphA-3xFLAG*
ME-872	CCCATCGTGATCTTTGTAGTCTCCCAGTGCGCCGATCACTTCAAGTTCTGTTAG	Plasmid construction, pRE112-*aphA-3xFLAG*
ME-873	GCACTGGGAGACTACAAAGATCACGATGGGGATT	Plasmid construction, pRE112-*aphA-3xFLAG*
ME-560	TTTGTCGTCGTCATCCTTGTAGTC	Plasmid construction, pRE112-*aphA-3xFLAG*
ME-874	ATATCGACTACAAGGATGACGACGACAAATAATTCGTCACTTTAAATAAAACGAAAAAGG	Plasmid construction, pRE112-*aphA-3xFLAG*
ME-875	ACGCCTGAATAAGTGATAGGGCCCGATCCCTTTGAGGAATTTTGATTTCGTGGTGG	Plasmid construction, pRE112-*aphA-3xFLAG*
ME-865	GGGTTGAGAAGCGGTGTAAGTGAACTGCATACTCAAAAGAGACCGTGGAAGC	Plasmid construction, pRE112-*3xFLAG-luxR*
ME-866	CTTGTAATCCCCATCGTGATCCTTGTAGTCCATATTTCTTTTTCCTTGCCATTTGAG	Plasmid construction, pRE112-*3xFLAG-luxR*
ME-867	GACTACAAGGATCACGATGGGGATT	Plasmid construction, pRE112-*3xFLAG-luxR*
ME-564	TCCCAGTGCTTTGTCGTCGTCATCCTTGTAGTC	Plasmid construction, pRE112-*3xFLAG-luxR*
ME-868	TACAAGGATGACGACGACAAAGCACTGGGAGACTCAATTGCAAAGAGACCTCG	Plasmid construction, pRE112-*3xFLAG-luxR*
ME-869	ACGCCTGAATAAGTGATAGGGCCCGATCCCAAGTATTTGAAGGCTCAATCACTGAC	Plasmid construction, pRE112-*3xFLAG-luxR*
ME-601	GTGAAGGGCAATCAGCTGTTG	Plasmid construction, transcriptional reporters in pFED343
ME-444	TCACTACTCTGTGCTATGGTGTTC	Plasmid construction, cloning in pFED343
ME-524	TGAGACGGGCAACAGCTGATTGCCCTTCACAAAAGTATACAGCATGGTTTGTGCC	Plasmid construction, P_{luxO}-*mRuby3*
ME-525	CTTAATCAATTCTTCACCCTTAGATACCATAAGTAGATAACGAGACTTTTGACCTTC	Plasmid construction, P_{luxO}-*mRuby3*
ME-526	ATGGTATCTAAGGGTGAAGAATTGATTA	Plasmid construction, P_{luxO}-*mRuby3* (*mRuby3* forward)
ME-527	GCATTGAACACCATAGCACAGAGTAGTGATTATTACTTATATAATTCATCCATTCCACCC	Plasmid construction, transcriptional reporters (*mRuby3* reverse)
ME-528	TGAGACGGGCAACAGCTGATTGCCCTTCACAAGTAGATAACGAGACTTTTGACCTTC	Plasmid construction, P_{qrr1}-*mRuby3*
ME-555	CCTAGGCCTGTCGAGGCTGTTTCCTGTGTGAAAAGTATACAGCATGGTTTGTGCC	Plasmid construction, P_{qrr1}-*mRuby3*
ME-554	CACACAGGAAACAGCCTCGAC	Plasmid construction, transcriptional reporters (rbs-*mRuby3* forward)

STR-0129	CTCGGGTCACCTATCCAACTGA	qRT-PCR, *qrr*1 (*V. harveyi)*
STR-0130	TCGGATCTATTGGCTCGTTCTG	qRT-PCR, *qrr*1 (*V. harveyi)*
STR-0131	CTTAAGCCGAGGGTCACCTAGC	qRT-PCR, *qrr*2 (*V. harveyi)*
STR-0132	CAATTAGGGCGATTGGCTTATGT	qRT-PCR, *qrr*2 (*V. harveyi)*
STR-0036	CTTAAGCCGAGGGTCACCTAGC	qRT-PCR, *qrr*3 (*V. harveyi)*
STR-0037	ACAAATTCGAGTCCACTAACAACGT	qRT-PCR, *qrr*3 (*V. harveyi)*
ME-251	GTTGATTGGCGGTATATACTTGTG	qRT-PCR, *qrr*4 (*V. harveyi)*
ME-252	CCTTATTAAGCCGAGGGTCAC	qRT-PCR, *qrr*4 (*V. harveyi)*
STR-0133	GACGTTGTTAGTGAACCCAATTGTT	qRT-PCR, *qrr*5 (*V. harveyi)*
STR-0134	CACAAGGTTTGTGATTGGCTGTATA	qRT-PCR, *qrr*5 (*V. harveyi)*
ME-566	TGAGACGGGCAACAGCTGATTGCCCTTCACCG GGTGAAGTTGCGAGTTTCA	Plasmid construction, P_{qrr2}-*mRuby3*
ME-567	CCTAGGCCTGTCGAGGCTGTTTCCTGTGTGAA AAGAATTATGCATTAATCATGCCAG	Plasmid construction, P_{qrr2}-*mRuby3*
ME-568	TGAGACGGGCAACAGCTGATTGCCCTTCACGT GTGCTGATCCCAATTGTTCTTG	Plasmid construction, P_{qrr3}-*mRuby3*
ME-569	CCTAGGCCTGTCGAGGCTGTTTCCTGTGTGCA CTAAATGATGCAGTTAGTGTGCC	Plasmid construction, P_{qrr3}-*mRuby3*
ME-570	TGAGACGGGCAACAGCTGATTGCCCTTCACTG ATGAAAATCGCCGATGAACG	Plasmid construction, P_{qrr4}-*mRuby3*
ME-571	CCTAGGCCTGTCGAGGCTGTTTCCTGTGTGAT CTGTATAAAGCACGATGCGT	Plasmid construction, P_{qrr4}-*mRuby3*
ME-572	TGAGACGGGCAACAGCTGATTGCCCTTCACCT ATCGAGACCGCATTGACAG	Plasmid construction, P_{qrr5}-*mRuby3*
ME-573	CCTAGGCCTGTCGAGGCTGTTTCCTGTGTGTT ACAACTAAAGCATTAGGCATGCC	Plasmid construction, P_{qrr5}-*mRuby3*
ME-1024	CAATTGTGGTTTCTTATGAAGTCCATAC	P_{luxC} EMSA probe 1 forward primer
ME-1025	TTTAAGTGGTTGCTGCTACTAGAG	P_{luxC} EMSA probe 1 reverse primer
ME-1026	CTCTAGTAGCAGCAACCACTTAAA	P_{luxC} EMSA probe 2 forward primer
ME-1027	CACTAAAGCAACCATACTCATAAATATTG	P_{luxC} EMSA probe 2 reverse primer

ME-1028	CAATATTTATGAGTATGGTTGCTTTAGTG	P_{luxC} EMSA probe 3 forward primer
ME-1029	TTATAATTAGTCATAACATTTAACAAACAACGAA	P_{luxC} EMSA probe 3 reverse primer
ME-1030	TTCGTTGTTTGTTAAATGTTATGACTAATTATAA	P_{luxC} EMSA probe 4 forward primer
ME-1031	TTTTAACCAGATTTATTAAGCAGATCAAAC	P_{luxC} EMSA probe 4 reverse primer
ME-1032	GTTTGATCTGCTTAATAAATCTGGTTAAAA	P_{luxC} EMSA probe 5 forward primer
ME-1033	TCCATATCAAGAGCTTCTCCTTTG	P_{luxC} EMSA probe 5 reverse primer
ME-1034	CAAAGGAGAAGCTCTTGATATGGA	P_{luxC} EMSA probe 6 forward primer
ME-1035	TTCTTCAAAACTGATCTCAAATCGATT	P_{luxC} EMSA probe 6 reverse primer
ME-443	GCTTAATTACCTCCTCTTCCTTAGCTCCTGAAT TCCTAG	Plasmid construction, overexpression constructs in pFED343
ME-636	CAGGAGCTAAGGAAGAGGAGGTAATTAAGCAT GGACTCAATTGCAAAGAGACCT	Plasmid construction, P_{tac}-*luxR*
ME-637	AGCATTGAACACCATAGCACAGAGTAGTGATTA GTGATGTTCACGGTTGTAGATG	Plasmid construction, P_{tac}-*luxR*
ME-620	GCTTAATTACCTCCTTCAGACCGCTTCTGCGTT C	Plasmid construction, P_{BAD}-*luxT*
ME-621	AGAATTTGCCTGGCGGCAG	Plasmid construction, P_{BAD}-*luxT*
ME-622	GCAGAAGCGGTCTGAAGGAGGTAATTAAGCAT GCCAAAGCGTAGTAAAGAAGATAC	Plasmid construction, P_{BAD}-*luxT*
ME-623	CCACCGCGCTACTGCCGCCAGGCAAATTCTTT ATTTGCTCATTTGAATTAACGAACG	Plasmid construction, P_{BAD}-*luxT*
ME-790	TTAAGCTGCAAGGGCAAATCG	qRT-PCR, *hfq* (*E. coli)*
ME-791	GGACAACAGTAGAAATCGCGTG	qRT-PCR, *hfq* (*E. coli)*
ME-128	GAAGATCATGGATGCCGTTGTTG	qRT-PCR, *luxT* (*V. harveyi)*
ME-415	AATGGTGGCTAATACCTGTACGC	qRT-PCR, *luxT* (*V. harveyi)*
ME-398	GGGTTGAGAAGCGGTGTAAGTGAACTGCATTT GTGAGCAAGGCGTTGACTTCGTAGC	Plasmid construction, *VIBHAR_RS03920* deletion
ME-399	GGTAGGACTAGACACAAGCAACC	Plasmid construction, *VIBHAR_RS03920* deletion
ME-400	TTCATGAGGTTGCTTGTGTCTAGTCCTACCCCT GCTTAATTATATCGCCCAATAG	Plasmid construction, *VIBHAR_RS03920* deletion

ME-401	ACGCCTGAATAAGTGATAGGGCCCGATCCCTT GTGCTCAGTTTAATGCTGGTG	Plasmid construction, *VIBHAR_RS03920* deletion
STR-0383	ACATCAACTCAAATGGCAAGG	qRT-PCR, *luxR* (*V. harveyi)*
STR-0384	GCAAACACTTCAAGAGCGATTT	qRT-PCR, *luxR* (*V. harveyi)*
STR-0381	ATCCATCAACTCTAGGTGATAAACG	qRT-PCR, *aphA* (*V. harveyi)*
STR-0382	CGTCGCGAGTGCTAAGTACA	qRT-PCR, *aphA* (*V. harveyi)*
ME-778	CGGCAACCAAAAAAGTGGTCG	qRT-PCR, *VIBHAR_RS11785*
ME-779	ACCCCATTGTTGGTTGTTCATGTTG	qRT-PCR, *VIBHAR_RS11785*
ME-782	GGCTATCATGGGGAGATCAAGTC	qRT-PCR, *VIBHAR_RS11620*
ME-783	GGTGATGGGCATTGAGACGTTAC	qRT-PCR, *VIBHAR_RS11620*
LF-495	AAACTGGCGCTTGATACAGG	qRT-PCR, *VIBHAR_RS16980*
LF-496	ACATTCTGCACCACTCGTTG	qRT-PCR, *VIBHAR_RS16980*
ME-780	AGACAACAGCTCCAATACGGC	qRT-PCR, *VIBHAR_RS25670*
ME-781	ATTTGGGTTGGCTTTGGTCTCTAC	qRT-PCR, *VIBHAR_RS25670*
LF-342	GAGTCGATGCCTCAAACCAC	qRT-PCR, *VIBHAR_RS26745*
LF-343	AGGAACTTCACCGAGTGTGT	qRT-PCR, *VIBHAR_RS26745*
LF-RT27	GACAGTGAAAAGTCTGGCCC	qRT-PCR, *VIBHAR_RS24795*
LF-RT28	TGACTTGCGCTTGGAAACTT	qRT-PCR, *VIBHAR_RS24795*
LF-RT55	TTGCTCTGAATGCCGCAAAT	qRT-PCR, *VIBHAR_RS18320*
LF-RT56	TGGCGCTCTTCTGATAGGTT	qRT-PCR, *VIBHAR_RS18320*
LF-772	CAAAACTGGCGACTGTCCAA	qRT-PCR, *VIBHAR_RS24765*
LF-773	TGCTTGCCAGATTCCCCTTA	qRT-PCR, *VIBHAR_RS24765*
LF-RT43	ACCCTTCTTGCTGCTTCTCT	qRT-PCR, *VIBHAR_RS26565*

LF-RT44	ACCAACACAATGGGATGCTG	qRT-PCR, *VIBHAR_RS26565*
YS-611	ACGAAGCACAGCGTATCATC	qRT-PCR, *VIBHAR_RS21890*
YS-612	TAGCAGCTGGCTCACTTCTT	qRT-PCR, *VIBHAR_RS21890*
LF-RT13	CGACCGATTGGAAAACGCTA	qRT-PCR, *VIBHAR_RS01950*
LF-RT14	ACGGTTGGCTATAACCTGCT	qRT-PCR, *VIBHAR_RS01950*
LF-RT19	AGTCCAAAGTATCGCTGAACA	qRT-PCR, *VIBHAR_RS11480*
LF-RT20	TACGTTGAACATCAGCCCCT	qRT-PCR, *VIBHAR_RS11480*
LF-497	TTTTGCTTCAACAGGCGCTA	qRT-PCR, *VIBHAR_RS18695*
LF-498	TGTCTATCTACGCATCGGCT	qRT-PCR, *VIBHAR_RS18695*
LF-RT1	CGTGAAGTCAGTCGTTTGGT	qRT-PCR, *VIBHAR_RS27840*
LF-RT2	GCATGTTCTGGATTTTGCGT	qRT-PCR, *VIBHAR_RS27840*
ME-82	CATTGCTTACCTCGCTCTCAG	qRT-PCR, *luxMN* (*V. harveyi)*
ME-83	GTATGGCGATAAGCCACTGATTAC	qRT-PCR, *luxMN* (*V. harveyi)*
ME-1042	GGGTTGAGAAGCGGTGTAAGTGAACTGCATTG TCGCTGGTGGCAATCTTG	Plasmid construction, pRE112-*luxO* D61E Δ*qrr*1
ME-1043	CTCGAGCAGAATAAGATCAGGAATG	Plasmid construction, pRE112-*luxO* D61E Δ*qrr*1
ME-1044	CATCGCATTCCTGATCTTATTCTGCTCGAGCTT CGTCTACCTGATATGACGG	Plasmid construction, pRE112-*luxO* D61E Δ*qrr*1
ME-1045	ACGCCTGAATAAGTGATAGGGCCCGATCCCAT TGGCGCACAACAGGCTG	Plasmid construction, pRE112-*luxO* D61E Δ*qrr*1
ME-447	CAGGAGCTAAGGAAGAGGAGGTAATTAAGCAT GCCAAAGCGTAGTAAAGAAGATAC	Plasmid construction, P_{tac}-*luxT*
ME-448	AGCATTGAACACCATAGCACAGAGTAGTGATTA TTTGCTCATTTGAATTAACGAACG	Plasmid construction, P_{tac}-*luxT*
ME-981	TGAGACGGGCAACAGCTGATTGCCCTTCACAG CCCGAGCATACTAGTGATG	Plasmid construction, P_{11785}-*lux*
ME-982	GCCTGTCGAGGCTGTTTCCTGTGTGAATTAAC CTTTTATTATGGTTGTGTATTTTCTTAT	Plasmid construction, P_{11785}-*lux*
ME-1121	TAGGAATTCAATTAGGAGGTAATTAAGCATGGA AAAACACTTACCTTTAATAATAAATGG	Plasmid construction, transcriptional *lux* reporters

ME-1137	GCATTGAACACCATAGCACAGAGTAGTGATTATTACAAATAAGCGAACGCGTCC	Plasmid construction, transcriptional *lux* reporters
ME-985	TGAGACGGGCAACAGCTGATTGCCCTTCACGCTCTGGGATAACGTCATTAAGTG	Plasmid construction, P_{11620}-*lux*
ME-986	CCTAGGCCTGTCGAGGCTGTTTCCTGTGTGGTCCTTTATTTTAATGATTGAGTTGGTGC	Plasmid construction, P_{11620}-*lux*
ME-987	TGAGACGGGCAACAGCTGATTGCCCTTCACCAACATACTGGTCGACATCCCAG	Plasmid construction, P_{16980}-*lux*
ME-988	CCTAGGCCTGTCGAGGCTGTTTCCTGTGTGGTACCTAAAAGTGTAACCATAGCCAG	Plasmid construction, P_{16980}-*lux*
ME-983	TGAGACGGGCAACAGCTGATTGCCCTTCACACTAATGGAATACAACAAGATATAAGTCAC	Plasmid construction, P_{25670}-*lux*
ME-984	CCTAGGCCTGTCGAGGCTGTTTCCTGTGTGGCAACATTATTTAGCAACGCGC	Plasmid construction, P_{25670}-*lux*
ME-1006	GATTAAGCATTGGTAACTGTCAGACC	Plasmid construction, p*tetA*-Kan
ME-1007	AGTTTGTAGAAACGCAAAAAGGCC	Plasmid construction, p*tetA*-Kan
ME-1008	ACGGATGGCCTTTTTGCGTTTCTACAAACTCCTGTTAAGTATCTTCCTGGCATC	Plasmid construction, p*tetA*-Kan
ME-1009	ACTTGGTCTGACAGTTACCAATGCTTAATCCACATGGTCCTTCTTGAGTTTGTAAC	Plasmid construction, p*tetA*-Kan
ME-976	ACTAGTTCTAGAGCGGCCG	Plasmid construction, P_{tetA} *mVenus* translational reporters
ME-944	CTGTTTTGGCGGATGAGAGAAG	Plasmid construction, P_{tetA} *mVenus* translational reporters
ME-993	CACCGCGGTGGCGGCCGCTCTAGAACTAGTGCATTTTTGCTACGAATATACACACATAAG	Plasmid construction, P_{tetA}-*11785'-'mVenus*
ME-785	AACTCCAGTGAAAAGTTCTTCTCCTTTACTGACCAGCGATAATAAAGTGACGTTTC	Plasmid construction, P_{tetA}-*11785'-'mVenus*
ME-640	AGTAAAGGAGAAGAACTTTTCACTGG	Plasmid construction, P_{tetA} *mVenus* translational reporters
ME-994	GAAAATCTTCTCTCATCCGCCAAAACAGTTATTATTTGTATAGTTCATCCATGCCATGTG	Plasmid construction, P_{tetA} *mVenus* translational reporters
ME-998	CACCGCGGTGGCGGCCGCTCTAGAACTAGTGAGTACGCCCGCTTTTAGGTCAAAA	Plasmid construction, P_{tetA}-*11620'-'mVenus*
ME-789	AACTCCAGTGAAAAGTTCTTCTCCTTTACTCGTCGATAAGAAAGAAAGTGCAAGC	Plasmid construction, P_{tetA}-*11620'-'mVenus*
ME-999	CACCGCGGTGGCGGCCGCTCTAGAACTAGTCAACCGGTTGCATTGTTCGTGAA	Plasmid construction, P_{tetA}-*16980'-'mVenus*
ME-990	AACTCCAGTGAAAAGTTCTTCTCCTTTACTCGCAGTACCTAAAAGTGTAACCATA	Plasmid construction, P_{tetA}-*16980'-'mVenus*
ME-997	CACCGCGGTGGCGGCCGCTCTAGAACTAGTATGCCCAATTAAATTATGGCGCGTT	Plasmid construction, P_{tetA}-*25670'-'mVenus*

ME-787	AACTCCAGTGAAAAGTTCTTCTCCTTTACTACT ACCTAGCTTTGTATAGTTGAAA	Plasmid construction, P_{tetA}-*25670'-'mVenus*
ME-1063	CTTCCTTAGCTCCTGAATTCCTAG	Plasmid construction, P_{tac}-*qrr*1
ME-1064	ACAGGCCTAGGAATTCAGGAGCTAAGGAAGGG ACCCCTCGGGTCACCTATC	Plasmid construction, P_{tac}-*qrr*1
ME-1065	AGCATTGAACACCATAGCACAGAGTAGTGACG AACAGTTAATTCTTCTCTAACCG	Plasmid construction, P_{tac}-*qrr*1
ME-624	GGGTTGAGAAGCGGTGTAAGTGAACTGCATTG TGTGAAACCCGCGATAAGC	Plasmid construction, *V. cholerae luxT* deletion
ME-625	CATGGTCAGGCTCTTTTCTAACG	Plasmid construction, *V. cholerae luxT* deletion
ME-626	GATTTGACGTTAGAAAAGAGCCTGACCATGAAT TGATTCTTCACCTTCTGCCTAC	Plasmid construction, *V. cholerae luxT* deletion
ME-627	ACGCCTGAATAAGTGATAGGGCCCGATCCC ATGCTCACCCTTGCCGATATG	Plasmid construction, *V. cholerae luxT* deletion
ME-903	GGGTTGAGAAGCGGTGTAAGTGAACTGCATCG CGCTATTGTCTGGTTCAG	Plasmid construction, *V. parahaemolyticus swrT* deletion
ME-904	CTTTGGCATACTTTAAGCTCTTCTC	Plasmid construction, *V. parahaemolyticus swrT* deletion
ME-905	ACAAAGAGAAGAGCTTAAAGTATGCCAAAGTG GTTGATTGGACGCTCGC	Plasmid construction, *V. parahaemolyticus swrT* deletion
ME-906	ACGCCTGAATAAGTGATAGGGCCCGATCCCGG AATCGTAACTGCGCTCATC	Plasmid construction, *V. parahaemolyticus swrT* deletion
ME-969	GGGTTGAGAAGCGGTGTAAGTGAACTGCATGC TTAGGTGAGTTCGATGTCTTAG	Plasmid construction, pRE112-*luxO* D61E (*V. parahaemolyticus)*
ME-970	CTCGAGAAGAATAAGATCTGAAATTCGGTG	Plasmid construction, pRE112-*luxO* D61E (*V. parahaemolyticus)*
ME-971	CACCGAATTTCAGATCTTATTCTTCTCGAGCTT CGTCTGCCTGATATGACG	Plasmid construction, pRE112-*luxO* D61E (*V. parahaemolyticus)*
ME-972	ACGCCTGAATAAGTGATAGGGCCCGATCCCGC GGCGGTGGCAACATATC	Plasmid construction, pRE112-*luxO* D61E (*V. parahaemolyticus)*
ME-1054	TGAGACGGGCAACAGCTGATTGCCCTTCACCC TCAACCATCAAAAGGTAACGAG	Plasmid construction, P_{qrr1}-*mRuby3* (*V. parahaemolyticus*)
ME-1055	CCTAGGCCTGTCGAGGCTGTTTCCTGTGTGCT AATATATCAGCATGCTTTATGCCA	Plasmid construction, P_{qrr1}-*mRuby3* (*V. parahaemolyticus*)
ME-962	GGGTTGAGAAGCGGTGTAAGTGAACTGCATTG AGCGTGTCGAAATTATACGTG	Plasmid construction, pRE112-*luxO* D55E (*A. fischeri*)
ME-963	CTCTAGTAACACAAGATCAGGGGTTC	Plasmid construction, pRE112-*luxO* D55E (*A. fischeri*)
ME-964	TTAAGAACCCCTGATCTTGTGTTACTAGAGTTG CGCCTGCCTGACATG	Plasmid construction, pRE112-*luxO* D55E (*A. fischeri*)
ME-965	ACGCCTGAATAAGTGATAGGGCCCGATCCCGG CAGCATGGATAATTCGACTTC	Plasmid construction, pRE112-*luxO* D55E (*A. fischeri*)

ME-1052	TGAGACGGGCAACAGCTGATTGCCCTTCACGC AGCAACGGAAGCAGTATC	Plasmid construction, P_{qrr1}-*mRuby3* (*A. fischeri*)
ME-1053	CCTAGGCCTGTCGAGGCTGTTTCCTGTGTGAT ATACCTATTGCAGGGAGCGTG	Plasmid construction, P_{qrr1}-*mRuby3* (*A. fischeri*)
ME-949	GGGTTGAGAAGCGGTGTAAGTGAACTGCATGG TGCTATGTATAAGGGTGACCG	Plasmid construction, pRE112-*luxO* D55E Δ*qrr*1 (*A. fischeri)*
ME-950	TCTGCTATAAAATCAATAACTAACTATTCAC	Plasmid construction, pRE112-*luxO* D55E Δ*qrr*1 (*A. fischeri)*
ME-951	TGAATAGTTAGTTATTGATTTTATAGCAGAATAT ACCTATTGCAGGGAGCGTG	Plasmid construction, pRE112-*luxO* D55E Δ*qrr*1 (*A. fischeri)*
ME-952	ACGCCTGAATAAGTGATAGGGCCCGATCCCCT AGCCAAGGGTCTCGGTTTG	Plasmid construction, pRE112-*luxO* D55E Δ*qrr*1 (*A. fischeri)*
ME-93	GTTAACGGGATCAAACTACAGGGAC	qRT-PCR, *hfq* (*A. fischeri)*
ME-94	AGTAGAAATCGCATGCTTGTATACC	qRT-PCR, *hfq* (*A. fischeri)*
ME-1090	AACAAGGCTATCTCCAGAAAAGC	qRT-PCR, *litR* (*A. fischeri)*
ME-1091	TCTGCAATATCAGCATGACCACC	qRT-PCR, *litR* (*A. fischeri)*
