# Supplementary material for: LuxT controls specific quorum-sensing-regulated behaviors in Vibrionaceae spp. via repression of qrr1, encoding a small regulatory RNA
Source: PLoS Genet. 2021 Apr 1;17(4):e1009336. doi: 10.1371/journal.pgen.1009336 (PMC8043402; doi:10.1371/journal.pgen.1009336)
Supplement: S3 Table — (PDF) [file pgen.1009336.s003.pdf]

S3 Table. Plasmids used in this study

Plasmid Name	Stock Name	Description	Origin, marker	Reference
pET15b	pET15b	Overexpression vector for protein purification	pBR322, Amp^R	Novagen
pET15b-*luxT-6xHis*	pME127	*luxT-6xHis* overexpression vector for protein purification, cloned in pET15b	pBR322, Amp^R	This study
pRE112	pRE112	allelic exchange vector harboring *sacB* as a counter-selectable marker	R6Kγ, Cam^R	[1]
pRE112-Δ*luxT*	pME12	*V. harveyi luxT* deletion construct in pRE112	R6Kγ, Cam^R	This study
pRE112-*aphA-3xFLAG*	pME146	*V. harveyi aphA-3xFLAG* allele exchange construct in pRE112	R6Kγ, Cam^R	This study
pRE112-*3xFLAG-luxR*	pME147	*V. harveyi 3xFLAG-luxR* allele exchange construct in pRE112	R6Kγ, Cam^R	This study
pFED343	pFED343	P_{tac} overexpression vector	P15A, Cam^R	[2]
P_{qrr1}-*mRuby3*	pME98	*V. harveyi qrr*1-*mRuby3* transcriptional reporter in pFED343 (excluding the P_{tac} promoter)	P15A, Cam^R	This study
P_{luxO}-*mRuby3*	pME96	*V. harveyi luxO-mRuby3* transcriptional reporter in pFED343 (excluding the P_{tac} promoter)	P15A, Cam^R	This study
P_{qrr2}-*mRuby3*	pME100	*V. harveyi qrr*2-*mRuby3* transcriptional reporter in pFED343 (excluding the P_{tac} promoter)	P15A, Cam^R	This study
P_{qrr3}-*mRuby3*	pME102	*V. harveyi qrr*3-*mRuby3* transcriptional reporter in pFED343 (excluding the P_{tac} promoter)	P15A, Cam^R	This study
P_{qrr4}-*mRuby3*	pME103	*V. harveyi qrr*4-*mRuby3* transcriptional reporter in pFED343 (excluding the P_{tac} promoter)	P15A, Cam^R	This study
P_{qrr5}-*mRuby3*	pME105	*V. harveyi qrr*5-*mRuby3* transcriptional reporter in pFED343 (excluding the P_{tac} promoter)	P15A, Cam^R	This study
p*luxCDABE*	pBB1	*V. harveyi luxCDABE* cloned in pLAFR (expressed from its native promoter)	oriV Tet^R	[3]
p*luxR*	pME125	*V. harveyi luxR* overexpression vector, cloned in pFED343	P15A, Cam^R	This study
pKP8-35	pKP8-35	P_{BAD} overexpression vector	pBR322, Amp^R	[4]

p*luxT*	pME109	*V. harveyi luxT* overexpression vector, cloned in pKP8-35	pBR322, Amp^R	This study
pRE112-Δ*VIBHAR_RS03920*	pME64	*V. harveyi VIBHAR_RS03920* deletion construct in pRE112	R6Kγ, Cam^R	This study
pRE112-*luxO* D61E Δ*qrr*1	pME148	*V. harveyi luxO* D61E Δ*qrr*1 allele exchange construct in pRE112	R6Kγ, Cam^R	This study
p*luxT*	pME69	*V. harveyi luxT* overexpression vector, cloned in pFED343	P15A, Cam^R	This study
P_{11785}-*lux*	pME188	*VIBHAR_RS11785-luxCDABE* transcriptional reporter in pFED343 (excluding the P_{tac} promoter)	P15A, Cam^R	This study
P_{11620}-*lux*	pME189	*VIBHAR_RS11620-luxCDABE* transcriptional reporter in pFED343 (excluding the P_{tac} promoter)	P15A, Cam^R	This study
P_{16980}-*lux*	pME190	*VIBHAR_RS16980-luxCDABE* transcriptional reporter in pFED343 (excluding the P_{tac} promoter)	P15A, Cam^R	This study
P_{25670}-*lux*	pME191	*VIBHAR_RS25670-luxCDABE* transcriptional reporter in pFED343 (excluding the P_{tac} promoter)	P15A, Cam^R	This study
pXB300	pXB300	P_{tetA} overexpression vector	pBR322, Amp^R	[5]
p*tetA*-Kan	pME149	P_{tetA} overexpression vector (Amp^R replaced with Kan^R in pXB300)	pBR322, Kan^R	This study
P_{tetA}-*11785'-'mVenus*	pME150	*VIBHAR_RS11785* translational *mVenus* reporter, expressed from the *tetA* promoter	pBR322, Kan^R	This study
P_{tetA}-*11620'-'mVenus*	pME151	*VIBHAR_RS11620* translational *mVenus* reporter, expressed from the *tetA* promoter	pBR322, Kan^R	This study
P_{tetA}-*16980'-'mVenus*	pME152	*VIBHAR_RS16980* translational *mVenus* reporter, expressed from the *tetA* promoter	pBR322, Kan^R	This study
P_{tetA}-*25670'-'mVenus*	pME153	*VIBHAR_RS25670* translational *mVenus* reporter, expressed from the *tetA* promoter	pBR322, Kan^R	This study
p*qrr*1	pME154	*V. harveyi qrr*1 overexpression vector, cloned in pFED343	P15A, Cam^R	This study
pRE112-Δ*luxT* (*V. cholerae)*	pME112	*V. cholerae luxT* deletion construct in pRE112	R6Kγ, Cam^R	This study
P_{qrr1}-*luxCDABE*	pBK1001	*qrr*1-*luxCDABE* promoter fusion	Cam^R	[6]
pRE112-Δ*swrT* (*V. parahaemolyticus)*	pME155	*V. parahaemolyticus swrT* deletion construct in pRE112	R6Kγ, Cam^R	This study

pRE112-*luxO* D61E (*V. parahaemolyticus)*	pME156	*V. parahaemolyticus luxO* D61E allele exchange construct in pRE112	R6Kγ, Cam^R	This study
P_{qrr1}-*mRuby3* (*V. parahaemolyticus*)	pME157	*V. parahaemolyticus qrr*1-*mRuby3* transcriptional reporter in pFED343 (excluding the P_{tac} promoter)	P15A, Cam^R	This study
pRE112-*luxO* D55E (*A. fischeri)*	pME158	*A. fischeri luxO* D55E allele exchange construct in pRE112	R6Kγ, Cam^R	This study
P_{qrr1}-*mRuby3* (*A. fischeri*)	pME159	*A. fischeri qrr*1-*mRuby3* transcriptional reporter in pFED343 (excluding the P_{tac} promoter)	P15A, Cam^R	This study
pRE112-*luxO* D55E Δ*qrr*1 (*A. fischeri)*	pME160	*A. fischeri luxO* D55E Δ*qrr*1 allele exchange construct in pRE112	P15A, Cam^R	This study

Table S3 References

1. Edwards RA, Keller LH, Schifferli DM. Improved allelic exchange vectors and their use to analyze 987P fimbria gene expression. Gene. 1998 Jan 30;207(2):149–57.

2. Swem LR, Swem DL, Wingreen NS, Bassler BL. Deducing receptor signaling parameters from in vivo analysis: LuxN/AI-1 quorum sensing in *Vibrio harveyi*. Cell. 2008 Aug 8;134(3):461–73.

3. Miller MB, Skorupski K, Lenz DH, Taylor RK, Bassler BL. Parallel quorum sensing systems converge to regulate virulence in *Vibrio cholerae*. Cell. 2002 Aug 9;110(3):303–14.

4. Papenfort K, Pfeiffer V, Mika F, Lucchini S, Hinton JCD, Vogel J. SigmaE-dependent small RNAs of Salmonella respond to membrane stress by accelerating global *omp* mRNA decay. Mol Microbiol. 2006 Dec;62(6):1674–88.

5. Bina XR, Wong EA, Bina TF, Bina JE. Construction of a tetracycline inducible expression vector and characterization of its use in *Vibrio cholerae*. Plasmid. 2014 Nov;76:87–94.

6. Svenningsen SL, Waters CM, Bassler BL. A negative feedback loop involving small RNAs accelerates *Vibrio cholerae's* transition out of quorum-sensing mode. Genes Dev. 2008 Jan 15;22(2):226–38.
