# Supplementary material for: LuxT controls specific quorum-sensing-regulated behaviors in Vibrionaceae spp. via repression of qrr1, encoding a small regulatory RNA
Source: PLoS Genet. 2021 Apr 1;17(4):e1009336. doi: 10.1371/journal.pgen.1009336 (PMC8043402; doi:10.1371/journal.pgen.1009336)
Supplement: S1 Fig — EMSA showing binding of LuxT-6xHis to 95 bp DNA fragments containing the WT luxO promoter (left) and the luxO promoter in which the 50 nucleotides previously shown to be crucial for LuxT binding were randomized (right). DNA and protein concentrations as in Fig 2A. (PDF) [file pgen.1009336.s004.pdf]

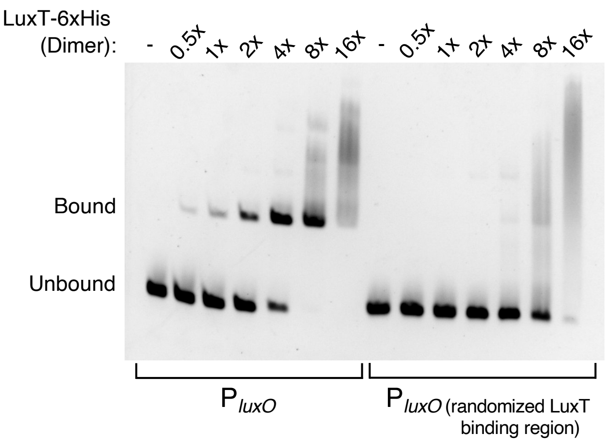
LuxT-6xHis
(Dimer):
-
0.5x
1x
2x
4x
8x
16x
-
0.5x
1x
2x
4x
8x
16x
Bound
Unbound
P luxO
P luxO (randomized LuxT binding region)
