# Supplementary material for: LuxT controls specific quorum-sensing-regulated behaviors in Vibrionaceae spp. via repression of qrr1, encoding a small regulatory RNA
Source: PLoS Genet. 2021 Apr 1;17(4):e1009336. doi: 10.1371/journal.pgen.1009336 (PMC8043402; doi:10.1371/journal.pgen.1009336)
Supplement: S2 Fig — The V. harveyi genomic DNA region harboring the LuxO-P, LuxT, and σ54 binding sites. The sites are labeled in relation to the qrr1 +1 transcriptional start site, which is also designated. Colors as in Fig 3A. (PDF) [file pgen.1009336.s005.pdf]

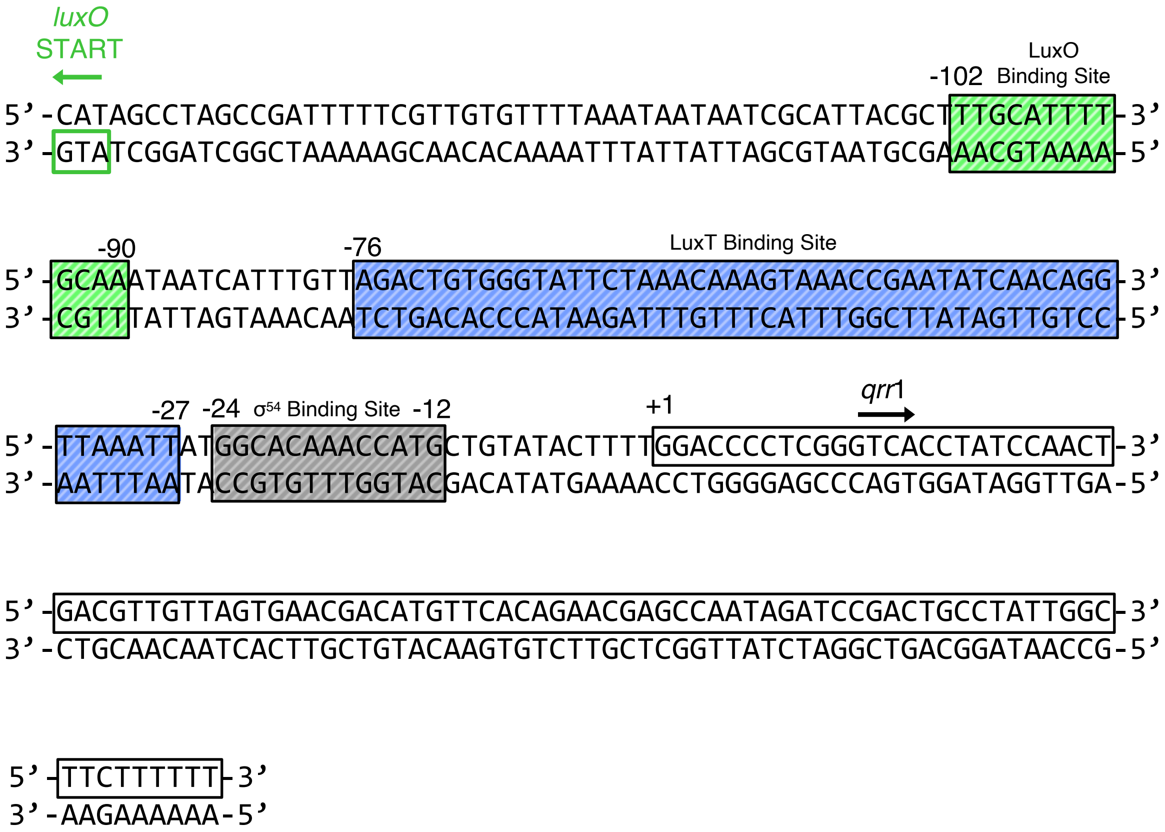

luxO
START
LuxO
Binding Site
-102
5’-CATAGCCTAGCCGATTTTTCGTTGTGTTTTAAATAATAATCGCATTACGCTTTGCATTTT-3’
3’-GTATCGGATCGGCTAAAAAGCAACACAAAATTTATTATTAGCGTAATGCGAAACGTAAAA-5’
-90
-76
LuxT Binding Site
5’-GCAAATAATCATTTGTTAGACTGTGGGTATTCTAAACAAAGTAAACCGAATATCAACAGG-3’
3’-CGTTTATTAGTAAACAATCTGACACCCATAAGATTTGTTTCATTTGGCTTATAGTTGTCC-5’
-27
-24
σ54 Binding Site
-12
+1
qrr1
5’-TTAAATTATGGCACAAACCATGCTGTATACTTTTGGACCCCTCGGGTCACCTATCCAACT-3’
3’-AATTTAATACCGTGTTTGGTACGACATATGAAAACCTGGGGAGCCCAGTGGATAGGTTGA-5’
5’-GACGTTGTTAGTGAACGACATGTTCACAGAACGAGCCAATAGATCCGACTGCCTATTGGC-3’
3’-CTGCAACAATCACTTGCTGTACAAGTGTCTTGCTCGGTTATCTAGGCTGACGGATAACCG-5’
5’-TTCTTTTTT-3’
3’-AAGAAAAAA-5’
