# Supplementary figures and images for: LuxT controls specific quorum-sensing-regulated behaviors in Vibrionaceae spp. via repression of qrr1, encoding a small regulatory RNA
Source: PLoS Genet. 2021 Apr 1;17(4):e1009336. doi: 10.1371/journal.pgen.1009336 (PMC8043402; doi:10.1371/journal.pgen.1009336)

A

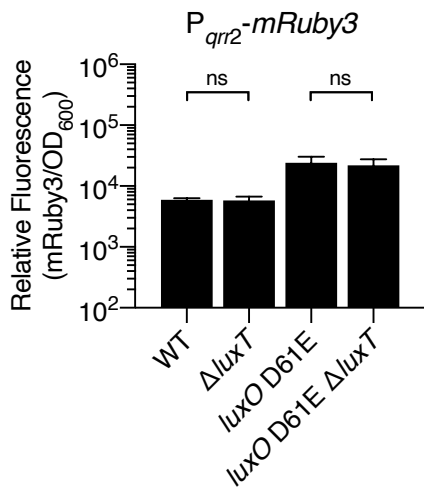

B

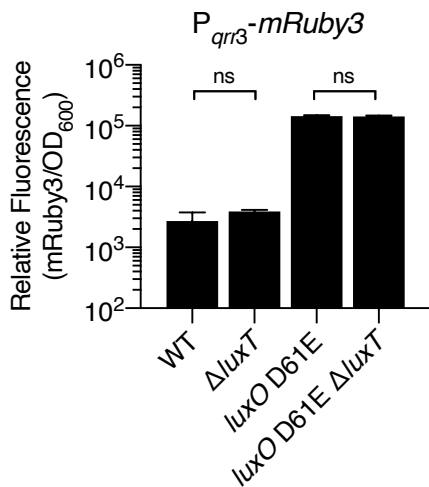

C

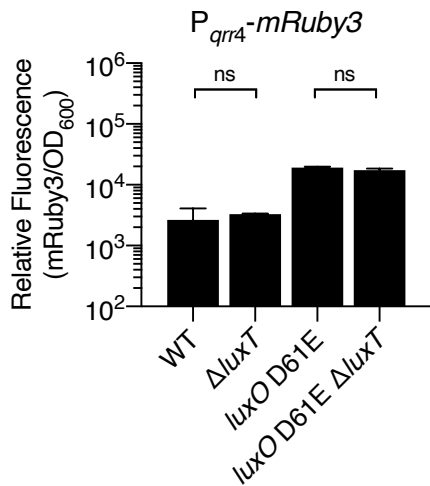

D

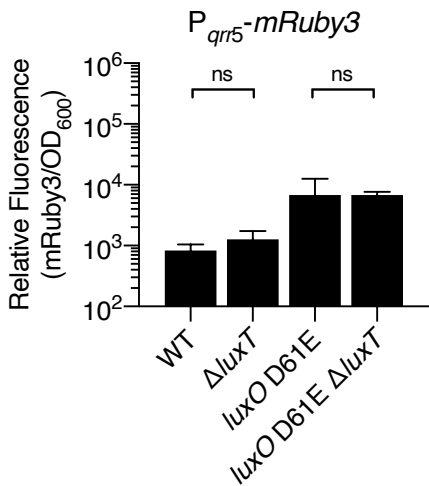

Supplement: S3 Fig — (A) Relative fluorescence values (mRuby3/OD600) of the indicated V. harveyi strains harboring a plasmid-borne Pqrr2-mRuby3 transcriptional reporter. Values represent relative fluorescence at OD600 = 0.6 for each sample. (B-D) As in A, except the strains harbor Pqrr3-mRuby3, Pqrr4-mRuby3, and Pqrr5-mRuby3, respectively. In all panels, error bars represent standard deviations of the means of n = 3 biological replicates. Unpaired two-tailed t tests with Welch’s correction were performed comparing the indicated two samples. p-values: ns ≥ 0.05. (PDF) [file pgen.1009336.s006.pdf]

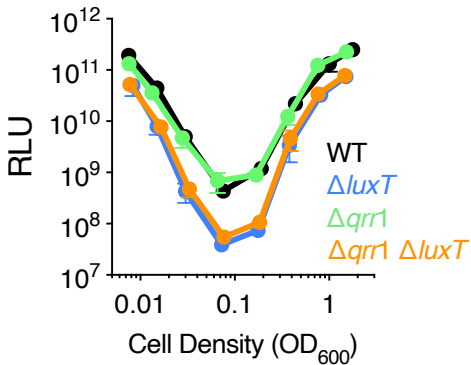

Supplement: S4 Fig — Density-dependent bioluminescence production from WT (black), ΔluxT (blue), Δqrr1 (green), and Δqrr1 ΔluxT (orange) V. harveyi strains. Relative light units (RLU) are counts/min mL-1 per OD600. Error bars represent standard deviations of the means of n = 3 biological replicates. (PDF) [file pgen.1009336.s007.pdf]

A

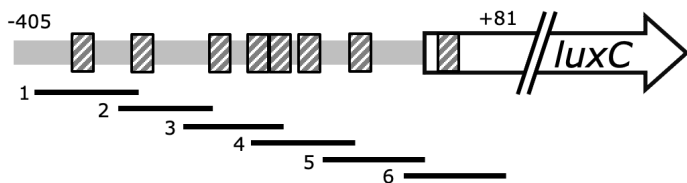

B

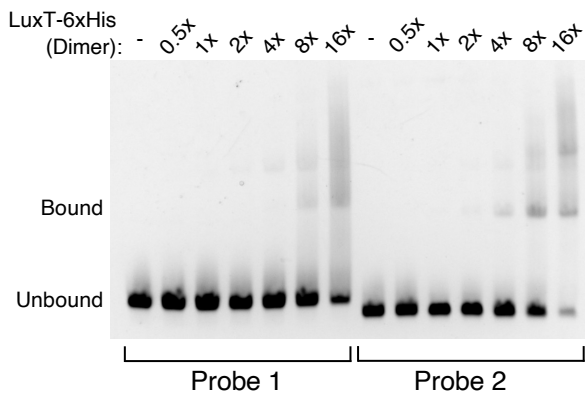

C

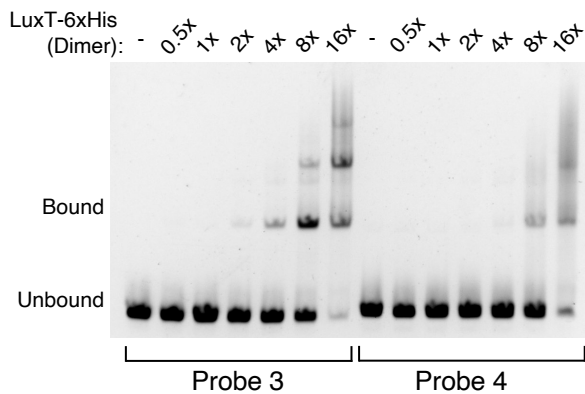

D

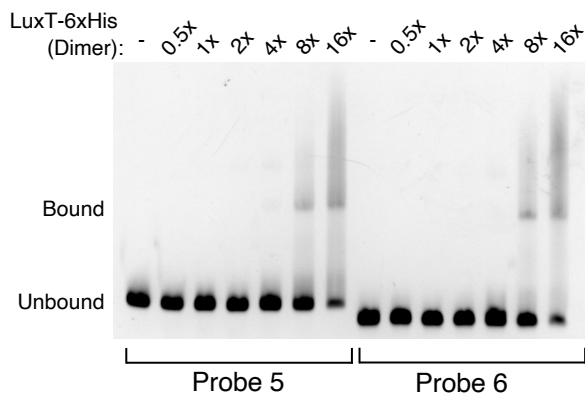

Supplement: S5 Fig — (A) Diagram of the luxCDABE promoter region. Black striped boxes represent known LuxR binding sites [34]. The black lines labeled 1 to 6 show the ~100 bp overlapping DNA fragments that were amplified and used as probes. The probes span the region -405 to +81 relative to the luxC start codon. (B-D) EMSAs measuring LuxT-6xHis binding to Probes 1–6 from panel A. DNA and protein concentrations as in Fig 2A. (PDF) [file pgen.1009336.s008.pdf]

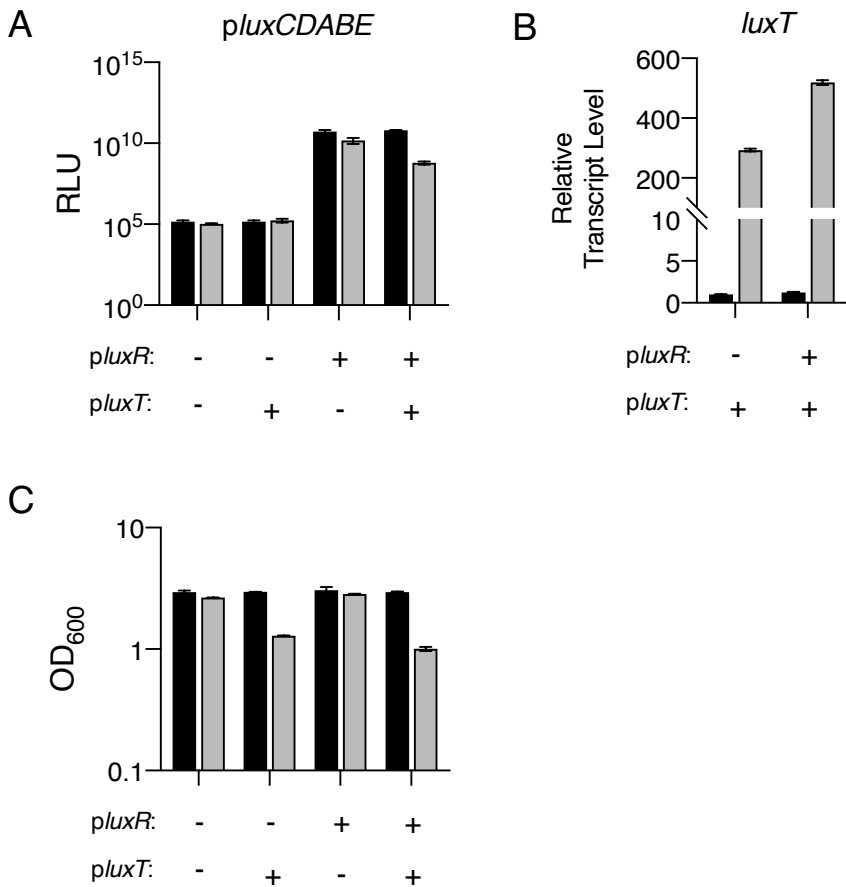

Supplement: S6 Fig — (A) Bioluminescence production from E. coli BW25113 harboring luxCDABE expressed from its native promoter on a plasmid (pBB1). The E. coli carries two additional plasmids, as indicated. - denotes the empty parent vector. + denotes the pluxR and/or the pluxT plasmid, encoding IPTG inducible luxR and arabinose inducible luxT, respectively. Strains were grown for 16 h in LB containing 0.5 mM IPTG in the absence (black) or presence (gray) of 0.2% arabinose. Relative light units (RLU) are counts/min mL-1 per OD600. (B) qRT-PCR measurements of luxT transcript levels in the E. coli strains harboring the pluxT plasmid from panel A. (C) Cell densities (OD600) of the strains in panel A after 24 h of growth. For panels B and C, the labeling and color schemes are as in panel A. In all panels, error bars represent standard deviations of the means of n = 3 biological replicates. (PDF) [file pgen.1009336.s009.pdf]

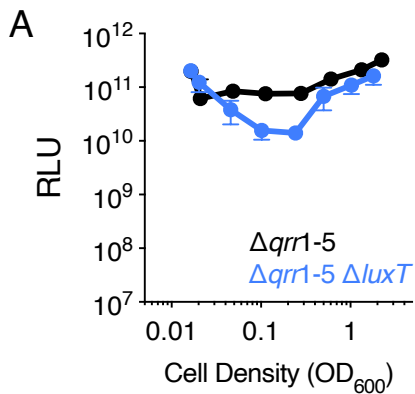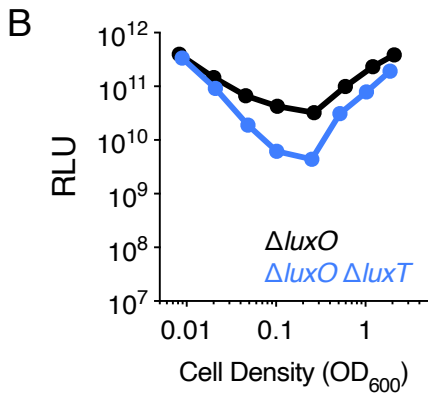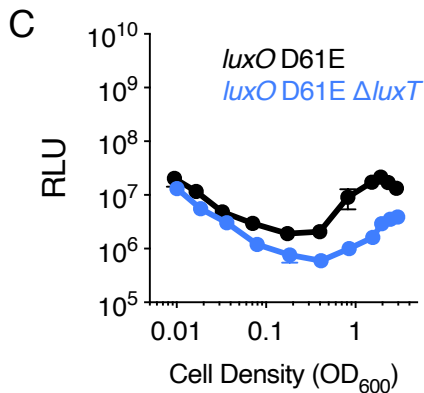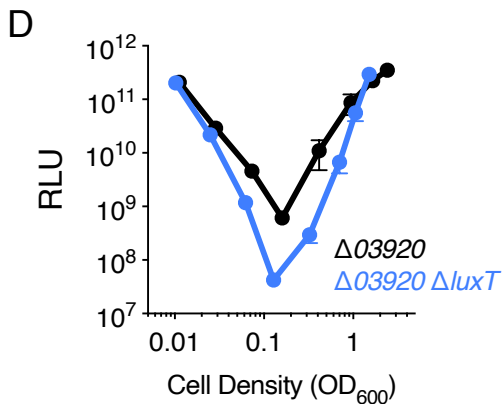

Supplement: S7 Fig — (A-D) Density-dependent bioluminescence production from the designated V. harveyi strains that possess (black) and lack (blue) luxT. Relative light units (RLU) are counts/min mL-1 per OD600. Error bars represent standard deviations of the means of n = 3 biological replicates. (PDF) [file pgen.1009336.s010.pdf]

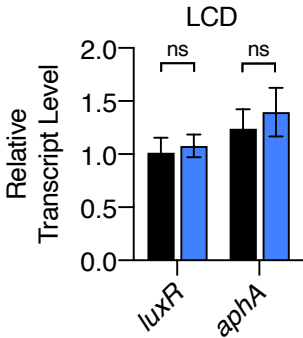

Supplement: S8 Fig — qRT-PCR measurements of luxR and aphA transcript levels in WT (black) and ΔluxT (blue) V. harveyi at LCD (OD600 = 0.05). Error bars represent standard deviations of the means of n = 3 biological replicates. Unpaired two-tailed t tests with Welch’s correction were performed comparing WT to ΔluxT. p-values: ns ≥ 0.05. (PDF) [file pgen.1009336.s011.pdf]

A

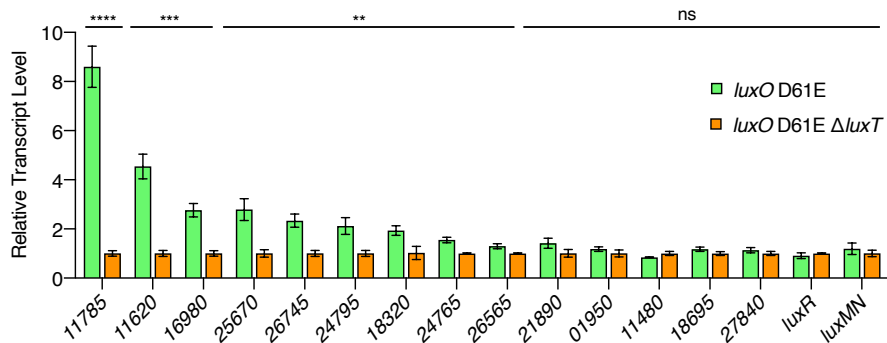

B

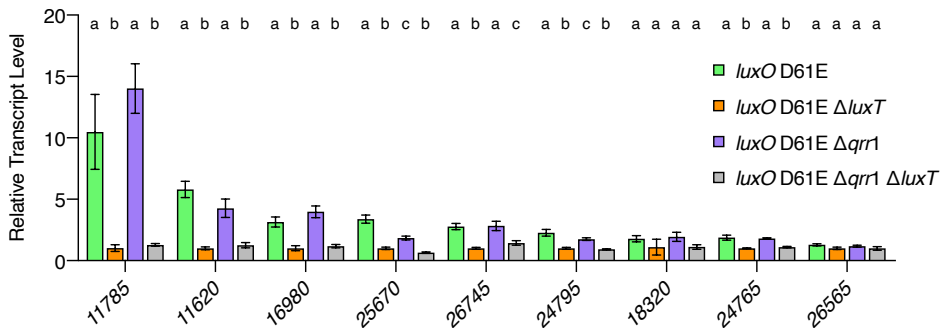

Supplement: S9 Fig — (A) Transcript levels of the indicated VIBHAR_RS genes as measured by qRT-PCR in V. harveyi luxO D61E and V. harveyi luxO D61E ΔluxT strains at OD600 = 1. Unpaired two-tailed t tests with Welch’s correction were performed comparing V. harveyi luxO D61E to V. harveyi luxO D61E ΔluxT. p-values: ns ≥ 0.05, ** < 0.01, *** < 0.001, **** < 0.0001. (B) qRT-PCR measurements of transcript levels of the indicated VIBHAR_RS genes in the designated V. harveyi strains at OD600 = 1. Different letters indicate significant differences between strains, p < 0.05 (two-way analysis of variation (ANOVA) followed by Tukey’s multiple comparisons test). In both panels, error bars represent standard deviations of the means of n = 3 biological replicates. (PDF) [file pgen.1009336.s012.pdf]

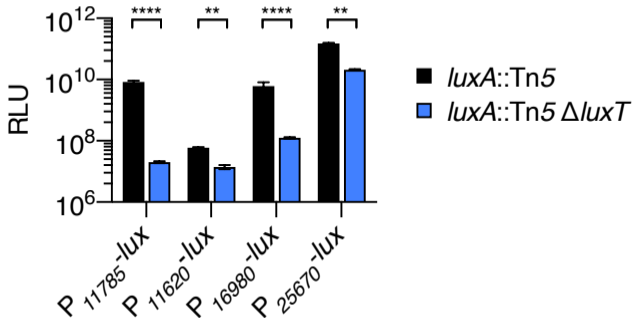

Supplement: S10 Fig — Activities of lux transcriptional fusions to the indicated promoters were measured in the designated V. harveyi strains at OD600 = 1. Relative light units (RLU) are counts/min mL-1 per OD600. Error bars represent standard deviations of n = 3 biological replicates. Unpaired two-tailed t tests with Welch’s correction were performed comparing V. harveyi luxA::Tn5 to V. harveyi luxA::Tn5 ΔluxT. p-values: ** < 0.01, **** < 0.0001. (PDF) [file pgen.1009336.s013.pdf]

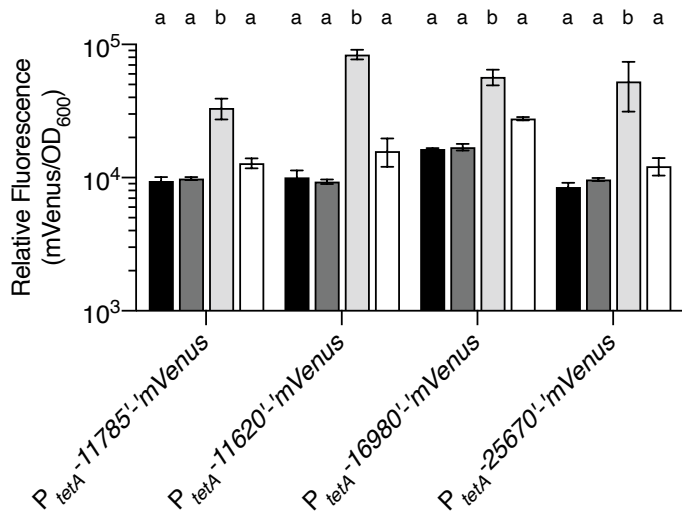

- aTc pControl
- aTc *pqrr1*
- + aTc pControl
- + aTc *pqrr1*

Supplement: S11 Fig — Relative fluorescence (mVenus/OD600) of WT V. harveyi harboring a plasmid encoding a translational reporter to the indicated VIBHAR_RS gene transcribed from the aTc inducible tetA promoter. The V. harveyi strains also carry IPTG-inducible qrr1 on a plasmid (pqrr1) or the empty parent vector (pControl). All strains were grown in the presence of 0.5 mM IPTG. Strains were grown in the absence and presence of 100 ng mL-1 aTc (- aTc and + aTc, respectively). Values represent relative fluorescence at OD600 = 0.3 for each sample. Error bars represent standard deviations of the means of n = 3 biological replicates. Different letters indicate significant differences between strains, p < 0.05 (two-way analysis of variation (ANOVA) followed by Tukey’s multiple comparisons test). (PDF) [file pgen.1009336.s014.pdf]

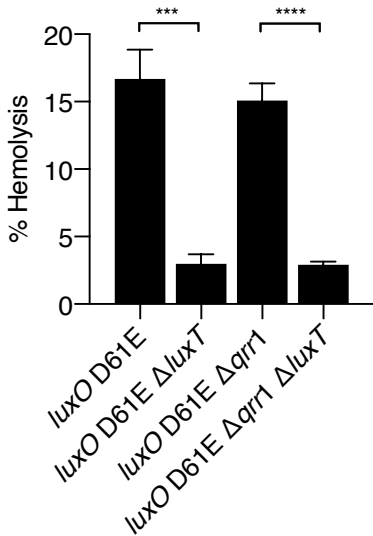

Supplement: S12 Fig — Hemolytic activity present in the indicated V. harveyi cell-free culture fluids as judged by lysis of defibrinated sheep’s blood. Culture fluids were collected after 24 h of growth in AB medium. Hemolytic activity was normalized to the activity of ddH2O [A415(sample)/A415(ddH2O) x 100]. Error bars represent standard deviations of the means of n = 3 biological replicates. Unpaired two-tailed t tests with Welch’s correction were performed comparing two samples, as indicated. p-values: *** < 0.001, **** < 0.0001. (PDF) [file pgen.1009336.s015.pdf]

**A**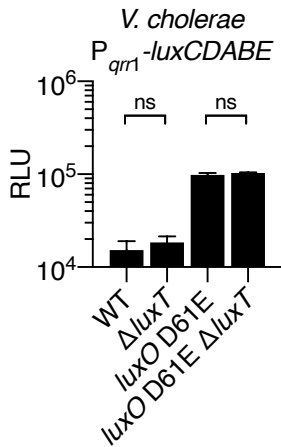**B**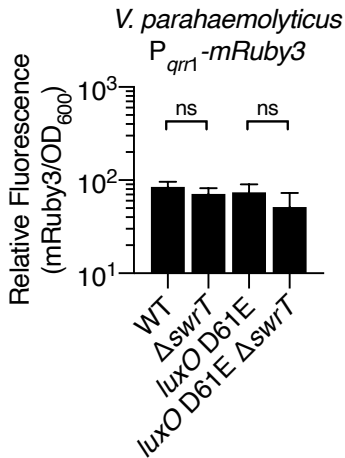

Supplement: S13 Fig — (A) Activity of a V. cholerae Pqrr1-luxCDABE transcriptional reporter in the indicated V. cholerae strains. (B) Relative fluorescence of a V. parahaemolyticus Pqrr1-mRuby3 transcriptional reporter measured in the indicated V. parahaemolyticus strains. Relative light production (panel A) and relative fluorescence (panel B) represent values when OD600 = 0.6 for each sample. Error bars represent standard deviations of the means of n = 3 biological replicates. Unpaired two-tailed t tests with Welch’s correction were performed comparing two samples, as indicated. p-values: ns ≥ 0.05. (PDF) [file pgen.1009336.s016.pdf]
