# Supplementary material for: LuxT controls specific quorum-sensing-regulated behaviors in Vibrionaceae spp. via repression of qrr1, encoding a small regulatory RNA
Source: PLoS Genet. 2021 Apr 1;17(4):e1009336. doi: 10.1371/journal.pgen.1009336 (PMC8043402; doi:10.1371/journal.pgen.1009336)
Supplement: Attachment — Submitted filename: LuxT qrr1 Response to Reviewers_final.docx [file pgen.1009336.s019.docx]

Dear Dr. Blokesch,

Thank you for handling our manuscript. We appreciate the reviewers’ comments and your guidance about how best to revise the paper. We are delighted that the referees found the topics we covered to be of interest. The two reviewers kindly provided us suggestions to improve our manuscript. We have taken both sets of suggestions to heart and revised the manuscript exactly as requested. Most notably, we cloned and assessed transcriptional reporter fusions to confirm the aspect of our model in which LuxT regulates target genes at the transcriptional level. We have also revised the text in all areas suggested by the referees.

A point-by-point list of our responses to the two referees is provided below. The referees’ comments are in black text and our responses are in blue text.

We hope you now find the manuscript acceptable for *PLOS Genetics*. Please let us know if you need anything more.

Yours,

Bonnie Bassler

*Reviewer #1:*

*In this manuscript submitted to PLOS Genetics, Eickhoff and colleagues describe the molecular mechanism by which the transcription factor LuxT regulates the expression of quorum sensing regulated genes via the Qrr1 sRNA in Vibrio harveyi. The five Qrr sRNAs were previously shown to regulate gene expression in this organism by base-pairing with complementary sequences in target mRNAs altering their stability or translation efficiency. Most, but not all of the target mRNAs identified so far are similarly regulated by all five Qrr sRNAs. In this manuscript under review, the authors provide evidence that the expression of Qrr1 sRNA is regulated differently than the other four Qrr sRNAs. Specifically, the authors show that LuxT represses transcription of qrr1 (Fig. 2) and that LuxT reduces the levels of Qrr1 sRNA, but not Qrr2, Qrr3, Qrr4, or Qrr5 (Fig. 3). The authors then provide evidence that LuxT increases expression of the luciferase genes by a mechanism that is independent of Qrr1 (Figs 2 and S4) and activates expression of 9 other genes by a mechanism that is dependent (Fig. 5 and S10) and independent (Fig. 4 and S9) of Qrr1. The authors then assess the conservation of transcriptional repression of qrr1 by LuxT homologs among several related species (Vibrio cholerae, Vibrio parahaemolyticus, Aliivibrio fischeri; Figs 6, 7, S12).*

*Overall, this manuscript is well written and well thought out and clearly demonstrates that Qrr1 is regulated by a mechanism distinct from the other Qrr sRNAs, i.e., via LuxT repression, and that LuxT regulates many quorum sensing-associated genes via a Qrr1 dependent and independent mechanism. Furthermore, this manuscript was a pleasure to read. A major sticking point for me was a central tenet put forth by the authors that LuxT activates the transcription of luxCDABE, VIBHAR_RS11785, VIBHAR_RS11620, VIBHAR_ RS16908, and VIBHAR_RS25670. While the authors show that the levels of these transcripts do increase in the absence of LuxT by a mechanism that is independent of the Qrr sRNAs, this increase could be due to LuxT indirectly regulating the stability of these transcripts, e.g., through another sRNA. In my opinion, the authors could resolve this conundrum by revising the text to make clear that LuxT activation of the transcription of these genes is only one possibility and to present alternative explanations supported by their results (minor revision) or to clearly show that LuxT activates the transcription of these genes using transcriptional fusions to their promoters (major revision). Other minor issues are also described below.*

*Major criticisms:*

1. *The authors indicate several times that LuxT activates the transcription of genes including VIBHAR_RS11785, VIBHAR_RS11620, VIBHAR_RS16908, and VIBHAR_RS25670, but this conclusion is not necessarily supported by the data. It is true that the levels of these transcripts go down in a luxT deletion strain regardless of whether qrr1 is present. However, the authors' experiments do not rule out the possibility that LuxT could cause an increase in the level of these transcripts by increasing the stability of these mRNAs, e.g., through some other sRNA. To decisively demonstrate that LuxT activates the transcription of these genes either directly or indirectly, the authors could test the impact of the luxT deletion on transcriptional reporter fusions to the promoters of these genes in the presence or absence of qrr1.*

As suggested by the referee, we tested our hypothesis that LuxT regulates these genes at the transcriptional level. To do this, we engineered transcriptional *lux* reporter fusions to *VIBHAR_RS11785, VIBHAR_RS11620, VIBHAR_RS16980*, and *VIBHAR_RS25670*. Specifically, the promoters of the four genes were cloned upstream of *luxCDABE* and a consensus ribosome-binding site was included to drive translation. Reporter activities were measured in *luxA*::Tn*5* and *luxA*::Tn*5* Δ*luxT V. harveyi* strains. Thus, any light produced comes from the reporter fusions. Indeed, LuxT activated transcription of all four fusions, i.e., light output was higher in the *luxA*::Tn*5* strain than in the *luxA*::Tn*5* Δ*luxT* strain. The following text was added to the manuscript along with a new S10 Fig showing the transcriptional fusion data:

“To demonstrate that LuxT control of these genes is exerted at the level of transcription, we made *lux* transcriptional reporters and measured their outputs in *luxA*::Tn*5* and *luxA*::Tn*5* Δ*luxT V. harveyi* strains. Using a *luxA* null mutant for this analysis ensured that all light production came from the transcriptional fusions. All four reporters exhibited lower activity in the *luxA*::Tn*5* Δ*luxT* strain than in the *luxA*::Tn*5* strain (~400, 4, 48, and 7-fold lower activity for, respectively, *VIBHAR_RS11785, VIBHAR_RS11620, VIBHAR_RS16980,* and *VIBHAR_RS25670*, S10 Fig).”

*Minor points:*

2. *Lines 394-396. The authors state based on Fig. 5E that deletion of qrr1 in the luxO D61E background strain caused increased hemolytic activity, but that result is not consistent with the quantitative assay of hemolysis (S11). The authors gloss over this fact.*

We thank the referee for noticing this difference between the results of the plate-based hemolysis assay and the liquid-based hemolysis assay that we had not addressed. We believe this discrepancy is due to the different growth conditions required for the two assays. Significant zones of clearing on the TSA blood agar plates did not appear until after ~72 h of growth, whereas the liquid assay measured activity in culture fluids isolated from *V. harveyi* cells grown for 24 h. The liquid assay is apparently more sensitive than the plates.

To address this issue, in the revised text, the following sentence was added:

“The hemolysis activities of the identical strains were also quantified using a liquid assay (S12 Fig). Analogous results were obtained for the four strains, except that the *luxO* D61E strain exhibited a level of hemolytic activity similar to that of the *luxO* D61E Δ*qrr*1 strain. Possibly, this discrepancy is due to the different growth conditions used for the plate and liquid hemolysis assays.”

3. *Lines 51, 59, 69. The authors reiterate several times that the five Qrr sRNAs are redundant; however, the authors already know from their previous work (PMIDs 22229925, 23838640) that this statement is not entirely accurate.*

The referee is correct. The Qrr sRNAs are not exactly redundant. We should not have simplified. For accuracy, in the revised manuscript, we now write:

“The small RNAs largely function redundantly making it difficult to understand why *V. harveyi* requires five of them.”

4. *It would be useful to differentiate the section titles from the body of the text in the results section by putting them in bold face type.*

The section headings have been changed to bold face type.

5. *Fig. 2B. Error bars appear to be missing. If error bars are so small that they are not visible, the authors should specifically denote that.*

The legend to Figure 2 was edited to include the following sentence:

“Standard deviations that are smaller than the symbols are not shown.”

6. *Figs. 3. It would be useful if the bars in B and C were colored using the same scheme as in D.*

We thank the referee for this suggestion. The colors in Fig 3B and 3C were changed to match that in Fig 3D.

7. *Line 205. The authors conclusion that “LuxT is a LCD transcriptional activator of luxCDABE” is not necessarily supported by their data. LuxT could be affecting transcript levels by impacting mRNA stability rather than transcription. Instead, the authors could state that “LuxT activates luxCDABE expression”*

The sentence was changed as suggested.

8. *Line 263. Change “LuxT activates luxCDABE transcription” to “LuxT activates luxCDABE expression”. See prior point.*

The sentence was changed as suggested.

9. *Line 346. Replace “transcription” with “expression”. No clear data supporting an impact on transcription versus mRNA stability.*

Please see our response to this same referee’s question #1. As suggested, we tested the role of LuxT on transcription of the target genes using transcriptional reporter fusions.

10. *Lines 398 to 399, 536, 543. Present data does not necessarily support transcriptional regulation.*

Please see above points 1 and 9 and our above responses. We thank the referee for drawing attention to this issue which was weakly supported in the original manuscript. The data from the newly included transcriptional reporter assays verify that LuxT activates transcription of the target genes.

11. *Lines 412 to 413. Take out parentheses.*

As suggested, the parentheses were removed.

12. *Lines 553 to 556. Diversification of DNA sequence of LuxT binding regions does not necessarily signify differences in LuxT-mediated repression of qrr1 expression in different species. As the authors indicate earlier, LuxT specificity could co-evolve with its binding site upstream of qrr1.*

As suggested, we softened our statement as follows:

“More broadly, our phylogenetic analyses of the LuxT binding regions upstream of *Vibrionaceae qrr*1 genes show that this DNA sequence has diversified, and consistent with our results, may signify that LuxT represses only a subset of *qrr*1 genes.”

13. *Line 612. Isopropyl should not be capitalized.*

This correction was made.

14. *Lines 691 to 704. The authors should indicate use of NTC and NRT controls.*

The qRT-PCR control experiments were indeed conducted but were not described in the Materials and Methods section. As suggested by the referee, the following sentence was added:

“Control reactions were performed with samples lacking reverse transcriptase and with samples lacking cDNA templates.”

*Reviewer #2: This manuscript by Eickhoff, et al., investigates regulation of quorum sensing (QS) in Vibrio harveyi via transcriptional and post-transcriptional mechanisms. They identify LuxT (a TetR-family transcription factor) as a direct repressor of qrr1, encoding one of the five QS small RNAs in V. harveyi. There are several major findings in this study. First, LuxT appears to be a global regulator of QS and exerts control over this system via direct transcriptional control of Qrr1 production and indirect control of other QS genes, including the luciferase operon. Another layer of LuxT activity is transcriptional control of (at least a subset of) Qrr1 target genes, though it is unclear whether this is direct or indirect. The authors use a very nice set of genetic experiments to convincingly demonstrate that LuxT regulates Qrr1 target genes both transcriptionally (Qrr1-independent) and post-transcriptionally (Qrr1-dependent). They also show that LuxT is conserved among the Vibrionaceae but that LuxT-dependent repression of qrr1 is only conserved in a subset of species. The findings add an additional layer of complexity to Vibrio QS systems and suggest a mechanism by which different signals could feed into the system to independently control activities of individual Qrr sRNAs. The study is of high quality. The experiments have the appropriate controls and the logic and interpretation of results is easy to follow. While I’m convinced that LuxT has the capacity to control QS genes both transcriptionally and post-transcriptionally, the post-transcriptional Qrr1-dependent effects could only be observed in reporter strains where the transcriptional effects were negated. Thus, the physiological relevance of the dual-control remains a mystery because the transcriptional effects of LuxT are clearly dominant. Moreover, qrr1 is the only gene that could be convincingly demonstrated as a direct transcriptional target of LuxT, so the mechanism of the other transcriptional effects remains unclear. Nevertheless, this work provides a new and elegant example of signal integration into a complex regulatory system and interesting multi-level control of gene expression.

Other comments:*

1. *I’m not convinced that the data support the assertion in the title. The LuxT regulation of QS behaviors was mostly via the transcriptional, Qrr1-independent pathway.*

We thank the referee for this comment. We agree that the Qrr1-independent effects of LuxT on the QS-controlled target genes do mask the Qrr1-dependent effects. However, we do not think that makes the latter mechanism less important. Rather, the findings show that the second mechanism (post-transcriptional control) must occur subsequent to the first mechanism (transcriptional control). Thus, the order of events, not the importance of the events, is revealed by our experiments. Deletion of *luxT* can only reveal the LuxT transcriptional effect because LuxT post-transcriptional activity cannot occur if LuxT transcriptional activity has not already occurred. Please see S11 Fig: if Qrr1 is expressed, it represses translation of the target genes.

Thus, our view, and we hope the referee will agree, is that the title accurately summarizes the findings reported in this manuscript.

With regard to the role of LuxT as a global transcriptional regulator, we are currently conducting RNA-Seq and binding site analyses to deepen our understanding of the LuxT regulon and which targets are directly and indirectly regulated.

2. *Line 130-131: What is meant by “distinct production profiles”? The list with “>” symbols suggests overall abundance. Is it also timing?*

The ranked list refers to the overall abundance of the Qrr sRNAs and was meant to convey that each is produced at a different level. The timing of when the Qrr sRNAs are expressed does not vary. They are all expressed at LCD (Tu and Bassler, 2007)

As suggested by the referee, the following sentence was changed to avoid confusion:

“Indeed, while all the Qrr sRNAs are made at LCD, they exhibit distinct production profiles. Specifically, in order of highest to lowest expression: Qrr4 > Qrr2 > Qrr3 > Qrr1 > Qrr5 [20].”

3. *Lines 159-163: When the Qrr1 targets are introduced it would be helpful to state whether they are exclusively regulated by Qrr1 or if they are also regulated by other Qrr sRNAs and provide the reference for the paper that identified them as Qrr1 targets.*

We thank the referee for this suggestion. All known target mRNAs of Qrr2-5 are identical. Qrr1, because it lacks 9 bp that are present in Qrr2-5, fails to regulate *aphA* and two other target mRNAs (neither of which are included in our present analyses). Besides these three targets, Qrr1 regulates the same set of mRNAs as do Qrr2-5.

For clarification, the following sentences were added to the Introduction:

“Qrr2-5 regulate an identical set of target mRNAs [31]. Thus, the failure of Qrr1 to control one subset of mRNAs is the only functional difference known among the Qrr sRNAs.”

As suggested by the referee, the following sentence was edited for clarification and a reference to the original paper was included.

“As a consequence, Qrr1 is available to post-transcriptionally regulate its target genes, including a gene encoding an extracellular protease (*VIBHAR_RS11785*), a gene encoding a pore-forming aerolysin toxin (*VIBHAR_RS11620*), a gene encoding a chitin deacetylase (*VIBHAR_RS16980*), and a gene specifying a component involved in capsular polysaccharide secretion (*VIBHAR_RS25670*) [31].”

4. *It would be very helpful to provide the legends indicating what bar colors represent for all figures, similar to Fig. S9B. It allows the meaning of the data to be more immediately obvious without having to search for the figure legends.*

Throughout the manuscript, when colors are used in the bar graphs, black indicates WT and blue indicates Δ*luxT.* At the suggestion of Referee #1, colors were added to Fig 3B and 3C to match those in Fig 3D. In the interest of space, additional labels were not added, but the same colors are now used consistently throughout. The color code is provided in the legends.

As suggested by the referee, additional labels were added to S9A Fig.

5. *In Fig. S9, most of the targets do not appear to be regulated by Qrr1 at all – i.e., there is no difference between isogenic qrr1+ and delta qrr1 strains. I do understand the model in Fig. 4B that attempts to explain why a Qrr1 effect might be less apparent in the context of the transcriptional control of LuxT, but that implies that the LuxT effect on transcription is all or nothing. In other words, in the presence of LuxT, transcription would have to be so high that post-transcriptional “activation” (repressing the Qrr1 repressor) would be irrelevant, and in the absence of LuxT, transcripts would have to be at such low levels that further repression by Qrr1 could not be measured. Is there any evidence for this? It looks like the different targets are present at a range of concentrations (though I might be misjudging because the data are reported as relative transcript levels). Addressing this would go a long way toward addressing my primary concern about this manuscript.*

We thank the referee for this question. Prior to the current study, no role had been ascribed to endogenously produced Qrr1*. qrr*1 expression is low in WT *V. harveyi*, including at LCD. Indeed, if one deletes *qrr*1 and measures light production, the canonical readout for *V. harveyi* QS, no difference is observed compared to the WT (S4 Fig and Tu and Bassler, 2007). Thus, prior to the present work, only *qrr*1 overexpression had provided some insight into function.

Concerning the referee’s statement that “the LuxT effect on transcription is all or nothing”, please see our response to this same referee’s question #1. In brief, and with respect, LuxT effects are not all or none, but rather, they occur sequentially-- transcriptional and then post-transcriptional. In S9 Fig, we show that deletion of *luxT* only reveals the LuxT transcriptional effect because LuxT post-transcriptional activity cannot occur if LuxT transcriptional activity has not already occurred.

We agree with the referee’s interpretation of the data in S9B Fig that there are no significant differences in transcript levels of the measured genes between the isogenic *qrr*1^+^ and Δ*qrr*1 strains. Please see our response to the same referee’s question #6 below. We believe that the difference is due to both the low production of Qrr1 in the presence of LuxT and the post-transcriptional mRNA sequestration mechanism by which Qrr1 represses its target mRNAs.

6. *Related to comment above: when Qrr1 targets are put under control of a heterologous promoter (Fig. 5), the Qrr1 effect is immediately evident, and the fold change between qrr1+ and deltaqrr1 strains appears fairly substantial for at least a couple of the targets. This again makes me think we should be able to see at least modest Qrr1-dependent regulation for some of these targets in Fig S9B.*

The referee is correct that in Fig 5A-D a Qrr1-dependent effect is observed between the isogenic *qrr*1^+^ and Δ*qrr*1 strains which is consistent with Qrr1 functioning as a repressor of translation. And, again, the referee is correct, that the qRT-PCR data in S9B Fig shows no such Qrr1-dependent effect in the same strains. The two experiments differ in that that the translational reporters directly measure translation, and the qRT-PCR experiments measure mRNA levels. We believe the differences between the two results highlight the post-translational mRNA sequestration mechanism by which Qrr1 represses the target genes.

The Qrr sRNAs are known to use three different mechanisms to repress translation. The mechanisms include catalytic degradation of the mRNA target, coupled degradation of the mRNA target and the Qrr sRNA, and sequestration of the mRNA target (Feng *et al*., 2015). In the sequestration mechanism, translation is inhibited by the Qrr, but target mRNA levels do not change.

The Qrr target genes tested in S9 Fig and in Fig 5 were originally identified by microarray analyses in which particular *qrr* genes were overexpressed (Shao and Feng *et al.,* 2013). In that same manuscript, the authors showed little to no effect of Qrr1 overexpression on mRNA levels of *VIBHAR_RS11785, VIBHAR_RS11620, VIBHAR_RS16980,* and *VIBHAR_RS25670* (previously in the NCBI database as *VIBHAR_02509, VIBHAR_02474, VIBHAR_03626,* and *VIBHAR_06665,* respectively). By contrast, Qrr1 readily repressed translational reporters of these same four genes (Shao and Feng *et al.,* 2013).

Thus, we think that the difference between the qRT-PCR and translational reporter experiments indicate that Qrr1 regulates the target genes by an mRNA sequestration mechanism that does not significantly alter mRNA levels.

The following sentence was added to the manuscript to provide an explanation for the qRT-PCR results:

“We presume that this pattern cannot be observed in the qRT-PCR analyses (S9B Fig) because Qrr1 represses translation of target genes by a sequestration mechanism that does not significantly alter mRNA levels [26, 31].”

7. *Fig. 5E hints at a Qrr1-dependent increase in aerolysin production, but the difference between luxO D61E and luxO D61E delta qrr1 is not evident in the quantitated hemolysin Fig. S11. This should be clarified.*

See response to Referee #1’s question #2. We provide it again here for ease:

We thank the referee for noticing this difference between the results of the plate-based hemolysis assay and the liquid-based hemolysis assay that we had not addressed. We believe this difference is due to the different growth conditions required for the two assays. Significant zones of clearing on the TSA blood agar plates did not appear until after ~72 h of growth, whereas the liquid assay measured activity in culture fluids isolated from *V. harveyi* cells grown for 24 h. The liquid assay is apparently more sensitive than the plates.

To address this issue, in the revised text, the following sentence was added:

“The hemolysis activities of the identical strains were also quantified using a liquid assay (S12 Fig). Analogous results were obtained for the four strains, except that the *luxO* D61E strain exhibited a level of hemolytic activity similar to that of the *luxO* D61E Δ*qrr*1 strain. Possibly, this discrepancy is due to the different growth conditions used for the plate and liquid hemolysis assays.”
